# Prevalence of Newcastle disease virus in feces of free-range turkeys in Enugu, Nigeria

**DOI:** 10.14202/vetworld.2020.1288-1293

**Published:** 2020-07-08

**Authors:** Obianuju Nkiruka Okoroafor, Paul Chukwuemeka Animoke, Edmund Chidiebere Mbegbu, Chinwe Justina Aronu, John Anelom Nwanta, Boniface Anene, John Ositadimma Okoye

**Affiliations:** 1 Department of Veterinary Medicine, Faculty of Veterinary Medicine, University of Nigeria, Nsukka, Nigeria; 2 Department of Veterinary Physiology and Pharmacology, Faculty of Veterinary Medicine, University of Nigeria, Nsukka, Nigeria; 3 Department of Animal Health and Production; Faculty of Veterinary Medicine, University of Nigeria, Nsukka, Nigeria; 4 Department of Veterinary Public Health, Faculty of Veterinary Medicine, University of Nigeria, Nsukka, Nigeria; 5 Department of Veterinary Pathology and Microbiology, Faculty of Veterinary Medicine, University of Nigeria, Nsukka, Nigeria

**Keywords:** Newcastle disease, prevalence, turkeys, virus shedding

## Abstract

**Background and Aim:**

Newcastle disease (ND) virus of free-range turkeys may be linked to outbreaks of ND in backyard chickens seen during Harmattan in Enugu State in Southeast Nigeria. This study aimed to determine the prevalence of ND virus and (NDV) detect NDV in the feces of free-range, domestic turkeys in Enugu, Nigeria.

**Materials and Methods:**

A total of 569 serum and 569 cloacal swab samples were collected from adult turkeys in selected households that keep turkeys and chickens together in the study area. The serum samples were assayed for antibodies against NDV using the hemagglutination inhibition (HI) test, whereas the cloacal samples were subjected to virus detection using a hemagglutination (HA) test.

**Results:**

A total of 186 serum samples (32.7%) were positive for NDV and 383 (67.3%) were negative. Of the 186 NDV-positive serum samples, 138 (74.2%) had HI titers ≥ 8. The remaining 48 (25.8%) serum samples had HI titers <8. NDV was detected from the cloacal swabs of turkeys with NDV -positive serum samples.

**Conclusion:**

The turkeys in this study were not previously vaccinated with the NDV vaccine; thus, those with NDV -positive serum samples and virus shedding in their feces may be potential risks to chickens reared in the same households as well as on commercial farms in the area. Those turkeys with sera negative for NDV are regarded to be at risk if they encounter a virulent strain of NDV. Regular vaccination of turkeys against the NDV is advised, especially in backyard farms, where turkeys are reared together with chickens and other species of poultry.

## Introduction

Newcastle disease (ND) is the most devastating viral disease of poultry, especially in developing countries, where the majority of the flock is raised on an extensive management system [1]. The disease is caused by Newcastle disease virus (NDV), an avian orthoavulavirus 1 that belongs to the genus *orthoavulavirus*, in the subfamily *Avulavirinae*, and family *Paramyxoviridae* [2,3]. More than 200 species of birds have been reported susceptible to natural and experimental infection with NDV, and it seems probable that more may be susceptible [3]. Chickens are most susceptible; turkeys are less susceptible; and quails, geese, and ducks are more resistant [4-8]. The spread and threat of ND to the poultry industry have been attributed mainly to commercial poultry reared in intensively managed poultry systems. Recently, attention has been drawn to scavenging village poultry species when various strains and pathotypes of NDV were isolated from apparently healthy village chickens, ducks, and quails [9,10]. Echeonwu [11] reported the isolation of a highly velogenic strain of NDV from apparently healthy ducks in Nigeria, which has been incriminated in the ND field and experimental outbreaks in chickens associated with very high mortality and morbidity. Thus, domestic chickens and other poultry species have been incriminated as sources of the spread of NDV [12-14]. Similarly, the ND outbreak of 1984 in the United Kingdom was found to be due to unvaccinated birds consuming feed contaminated by feces of infected pigeons [15], and in Nigeria, ND outbreaks have been linked to farms that rear exotic birds with local chickens, turkeys, and other avian species [16]. This shows that the spread of NDV in ND outbreaks witnessed in Nigeria, especially during the Harmattan season (mid-November through March) [17], is linked to rural community settings where a combination of different species and breeds of poultry such as chickens, turkeys, Muscovy ducks, and pigeons is reared together in the same compound [18]. This is also the case in cities where exotic poultry such as ostriches, peacocks, geese, and mallard ducks are kept together in the same compound and on some poultry farms [19]. The corearing of different species of poultry can facilitate the introduction and spread of NDV among poultry species and breeds in Nigeria as suggested by other researchers [12,19].

In Enugu state of Southeast Nigeria, it is also a common practice for households and even commercial farms to keep turkeys alongside local and exotic poultry. Outbreaks of ND have been reported in our current study area in addition to other parts of Nigeria in both rural and city settings, especially during Harmattan [17], and it is characterized by severe loss of both commercial and local poultry. These outbreaks in chickens have been linked to other species such as the pigeon in Great Britain [15] and turkeys in Indonesia [20], but in Nigeria, there is limited information on the susceptibility of turkeys to NDV and the role played by turkeys in transmitting the virus to other species.

This study aimed to investigate the presence of ND antibodies in the sera and NDV in the feces of turkeys to provide information on the role of turkeys in the ND outbreaks commonly seen in both local and exotic chickens in Enugu state of Southeast Nigeria.

## Materials and Methods

### Ethical approval and informed consent

Ethical approval for this study was given by the University Committee on Medical and Scientific Research Ethics, University of Nigeria, Nsukka. Written and signed consent were given by the turkey keepers before the study was conducted.

### Study area

**Figure-1:**
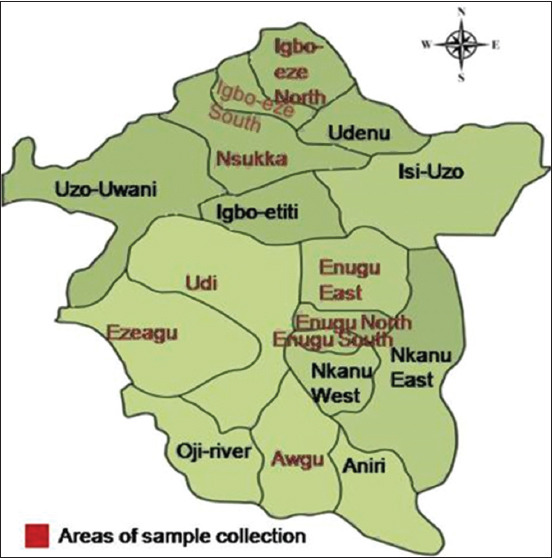
Map of Enugu state showing the areas of sample location.

The study was conducted in Enugu state, Southeast Nigeria (Figure-1). Enugu state is located between latitudes 5°56’N and 7°55’N and longitudes 6°53’E and 7°55’E. It covers a total land area of about 802,295 km^2^ and has a population of 2.5 million with a population density of 248 persons/square kilometer. The wet season lasts from April to October, while the dry season lasts from October to early April. The predominant poultry species kept is chickens (local and exotic), others are turkeys and ducks. These poultry species are reared intensively, semi-intensively, or on free-range system of management. Households that keep turkeys with other species and showed a willingness to be part of the study were purposively selected.

### Study period, location and study population

The study was conducted from March to October 2014. Adult turkeys kept in households that rear poultry, in randomly selected communities in the east, west, and north senatorial zones of Enugu state were chosen for this study. The turkeys selected had no previous history of NDV vaccination.

### Sample size, determination, and design

A total of 569 turkeys were sampled in the study area. The sample size was determined according to the previous methods [21] with a 95% confidence interval and 5% absolute precision.

### Sample collection

Turkeys are not routinely kept commercially in the study area; therefore, we were constrained to collect blood and cloacal samples from 20 adult turkeys per household visited. Blood samples were collected in sterile tubes without ethylenediaminetetraacetic acid (EDTA) and immediately sent to the department of veterinary medicine laboratory to harvest serum.

### Laboratory examinations

Blood (1 mL) was collected from each adult turkey sampled per household visited; serum was ­harvested and used in hemagglutination (HA) and hemagglutination inhibition (HI) tests. Cloacal swabs were collected from the same adult turkeys from which blood samples were collected and used for NDV detection using HA test. All fecal samples that showed HA were positive using known NDV antiserum [22].

### Statistical analysis

HI antibody titers >8 were considered positive [23]; thus, the numbers and percentages of turkeys at risk were calculated. Individual HI titers between the zones were compared by one-way ANOVA, and variant means were separated by Duncan’s multiple range test. Significance was accepted at p<0.05.

## Results

Of the 569 serum samples collected from adult turkeys in households that keep turkeys and chickens in Enugu state, 186 (32.7%) were positive for antibodies to NDV. Thus, 32.7% of the total serum samples collected from the three senatorial zones of Enugu state were positive, whereas 383 (67.3%) serum samples were negative for antibodies to NDV (Table-1). The mean ND HI antibody titer in free-range turkeys sampled in East Enugu (579.41±98.46) was significantly (p<0.05) higher than that of the west (61.89±11.69) and north (85.92±28.58) senatorial zones (Table-2).

**Table-1:** Prevalence of Newcastle disease virus antibodies in free-range turkeys in the three senatorial zones of Enugu state, Southeast Nigeria.

Senatorial zone	Local government area	No. of serum samples tested	No. of serum samples positive to ND antibodies (%)	No. of serum samples negative to ND antibodies (%)
Enugu East	EE, EN, ES	185	60 (32.4)	125(67.6)
Enugu West	UD, EZ, AG	175	53 (30.2)	122(69.8)
Enugu North	NS, IGE, IGZ	209	73 (34.9)	136(65.1)
	Total	569	186 (32.7)	383 (67.3)

EE=Enugu East, EN=Enugu North, ES=Enugu South, UD=Udi, EZ=Ezeagu, AG=Agwu, NS=Nsukka, IGE=Igbo-Etiti, IGZ=Igbo-Eze

**Table-2:** Distribution of mean HI antibody titer against Newcastle disease virus in free-range turkeys in the three senatorial zones of Enugu state, Southeast Nigeria.

Senatorial zone in Enugu state, Southeast Nigeria	Local government area	Number of serum samples tested	Mean HI titer
Enugu East	EE, EN, ES	185	579.41±98.46^a^
Enugu West	UD, EZ, AG	175	61.89±11.69^b^
Enugu North	NS, IGE, IGZ	209	85.92±28.58^b^
	Total	569	

EE=Enugu East, EN=Enugu North, ES=Enugu South, UD=Udi, EZ=Ezeagu, AG=Agwu, NS=Nsukka, IGE=Igbo-Etiti, IGZ=Igbo-Eze. Values with different superscripts; a,b,c within the column differ significantly (p≤ 0.05)

Of the 186 serum samples with detectable antibody titers against NDV, 138 (74.2%) had geometric mean HI titers ≥ 8 and were presumed to be protected against field challenge with NDV, whereas the remaining 48 (25.8%) serum samples had HI antibody titers < 8 and were presumed to be at risk (Table-3).

**Table-3:** Distribution of Newcastle disease HI antibody titers in turkeys reared in Enugu state, Southeast Nigeria.

Senatorial zones	Number of +ve sera	Titer	Titers
	
		<8	≥8
	
		Not protected	Protected
East Enugu	60	7	53
Enugu West	53	17	36
Enugu North	73	24	49
Total	186	48	138

HI titers ≥ 8 were considered protective. (OIE, 2012)

NDV was detected from the cloacal swabs of turkeys that had ND HI-positive sera in all of the households that kept both turkeys and other avian species together in the study area (Table-4).

**Table-4:** Virus detection from cloacal swab of turkeys sampled in Enugu state, Southeast Nigeria.

Senatorial zones	Local government area	HLD 1	HLD2	HLD3	HLD4	HLD5	HLD6	HLD7
Enugu East	EE	20/20	0/20	0/20	9/9	0/14	0/13	0/14
	EN	10/10	0/10	0/10	0/6	3/3	-	-
	ES	10/10	0/8	8/8	0/5	0/5	-	-
Enugu West	UD	0/15	10/10	0/12	0/4	9/9	-	-
	EZ	0/10	0/20	9/9	0/16	2/2	-	-
	AG	20/20	0/20	0/15	0/10	3/3	-	-
Enugu North	NS	20/20	20/20	0/20	0/20	0/20	0/20	0/16
	IGE	9/9	0/7	10/10	0/10	2/2	-	-
	IGZ	7/7	0/10	0/11	5/5	2/2	-	-

EE=Enugu East, EN=Enugu North, ES=Enugu South, UD=Udi, EZ=Ezeagu, AG=Agwu, NS=Nsukka, IGE=Igbo-Etiti, IGZ=Igbo-Eze, HLD=Household visited

## Discussion

Detection of ND hemagglutinating antibodies in unvaccinated turkeys sampled in the study area suggests that the turkeys may have been exposed to NDV either through the ingestion of contaminated feed or water, inhalation of the virus, contact with other infected birds, or contact with fomites [24]. In this study, turkeys that tested positive for ND may be those that recovered from an outbreak of ND or were purchased from other areas that had outbreaks because vaccination of village poultry is rarely undertaken [25]. These turkeys could act as reservoirs of NDV [14,24,26,27]. The detection of ND hemagglutinating antibodies in turkeys that were sampled in the present study and in a previous study in Kaduna state, Nigeria [28], suggests that circulation of NDV in turkeys in the southeastern and northern parts of Nigeria may be influenced by the movement of local poultry and poultry products from the north to the southeast and vice versa.

A seroprevalence of 32.7% in turkeys in Southeast Nigeria in the present study is at variance with 68%, 57.2%, and 53.7% recorded from turkeys in the Nigerian cities of Zaria (north-central), Maiduguri (northeastern), and Gombe (northern) [16,17,19], respectively. These observed regional differences in ND prevalence show an ecological variation in NDV activity and may be a reflection of the impact of the environment on the viability, spread, and epidemiology of NDV [29,30]. The lower seroprevalence value of 32.7% recorded in turkeys in Southeast Nigeria in comparison with 60.7% value recorded from local chickens previously sampled in the same zone [31] suggests that NDV infection in chickens is more severe than that in turkeys, and turkeys are likely to be sources of infection for other species, especially chickens.

The widespread presence of NDV antibodies in turkeys, as demonstrated in this study, implies that husbandry practices (backyard and commercial poultry) where different species of poultry, including turkeys are reared together may encourage cross-infection with NDV among the different species. This risk may be higher in areas where NDV vaccination is not commonly practiced as in the case of local or household poultry. From this study, 67.3% of the turkeys sampled that had no detectable antibodies against ND in addition to 25.9% of the turkeys with ND-positive serum and HI titers < 8 are presumed at risk and may not be protected against a challenge from a virulent strain of ND [23]. Researchers have reported that the risk of infection in such unprotected turkeys could be reduced by NDV vaccination [32-34].

The very high antibody titers against ND that we recorded in the east senatorial zone of Enugu when compared with the other two other zones are a reflection of the high density of poultry farms in East Enugu, which includes Enugu urban and neighboring towns and villages. It also implies that the epidemiology of the disease among zones varies with the highest chances of NDV transmission occurring in areas where other poultry species are reared with unvaccinated turkeys [17].

Detection of NDV in the feces of ND-positive turkeys sampled in the study area suggests that these turkeys appeared healthy or have recovered from a mild natural infection that induced a carrier status. This may constitute a potential threat to other species of poultry kept together with these turkeys and suggests that NDV may continually be present and circulate among poultry species in the study area. Furthermore, in our locality, poultry manure is commonly used by peasant farmers in the cultivation of farms which were local chickens and other farm animals scavenge for food. This manure often lodges in the undersides of footwear or the tires of automobiles and, by these means, NDV infection can spread even to commercial poultry farms. The possibility of this mode of spread could be one of the reasons for the sporadic outbreaks witnessed during the dry Harmattan season, which is shortly after the planting season in Nigeria. These sporadic outbreaks of ND occur with high morbidity and mortality, thereby affecting food security, which may result in malnutrition, unemployment, and great losses to backyard poultry keepers. It is, therefore, advised that all species of poultry be vaccinated against NDV.

## Conclusion

Detection of NDV in the serum and feces of turkeys in the Enugu state of Southeast Nigeria should discourage the practice of keeping different bird species together because it increases the chances of NDV transfer across species. Poultry farmers who keep turkeys should be encouraged to routinely vaccinate these birds against NDV.

## Authors’ Contributions

ONO conceived the work and conducted the field work with PCA, ECM, and CJA. JAN and BA designed and supervised the study. OON drafted the manuscript, interpreted data and CJA edited the manuscript. JOO reviewed the manuscript. All authors read and approved the final manuscript.
